# Successful Treatment of a Mixed Neuroendocrine-Nonneuroendocrine Neoplasm of the Colon with Metastases to the Thyroid Gland and Liver

**DOI:** 10.1155/2020/5927610

**Published:** 2020-02-13

**Authors:** Yuji Kanazawa, Masahiro Kikuchi, Yukihiro Imai, Nobuyuki Katakami, Satoshi Kaihara, Shogo Shinohara

**Affiliations:** ^1^Department of Otolaryngology, Head and Neck Surgery, Kobe City Medical Center General Hospital, 2-1-1 Minatojima Minami-machi, Chuo-ku, Kobe 650-0047, Japan; ^2^Department of Otolaryngology, Head and Neck Surgery, Graduate School of Medicine, Kyoto University, 54 Shogoin Kawahara-cho, Sakyo-ku, Kyoto 606-8507, Japan; ^3^Department of Clinical Pathology, Kobe City Medical Center General Hospital, 2-1-1 Minatojima Minami-machi, Chuo-ku, Kobe 650-0047, Japan; ^4^Department of Medical Oncology, Kobe City Medical Center General Hospital, 2-1-1 Minatojima Minami-machi, Chuo-ku, Kobe 650-0047, Japan; ^5^Department of Surgery, Kobe City Medical Center General Hospital, 2-1-1 Minatojima Minami-machi, Chuo-ku, Kobe 650-0047, Japan

## Abstract

Patients with mixed neuroendocrine-nonneuroendocrine neoplasms (MiNENs) of the colon have poor prognosis. Herein, we report a patient with MiNEN of the colon with metastases to the liver and the thyroid gland, with long-term survival. A 45-year-old man presented with anterior neck swelling. Histopathological examination of the thyroid tumor revealed neuroendocrine carcinoma (NEC), suggesting that a primary NEC in another organ had metastasized to the thyroid gland. Computed tomography to identify a primary NEC revealed two tumors: one in the liver and one in the transverse colon. A biopsy revealed that the histopathology of the liver and colon tumors was NEC and adenocarcinoma, respectively. Thereafter, the patient underwent surgical resection of the colon tumor and was finally diagnosed as colon MiNEN with metastases to the thyroid and liver. The surgical resection of the metastatic liver tumor was performed after several courses of systemic chemotherapy, and the patient survives presently without any recurrence for approximately seven years after the diagnosis. Surgical resection of each metastatic lesion combined with systematic chemotherapy apparently improved the prognosis of MiNEN of the colon with distant metastases.

## 1. Introduction

Neuroendocrine neoplasms of the colon are a rare but life-threatening malignancy [1]. The World Health Organization (WHO) classifies high-grade neuroendocrine neoplasms into two types: neuroendocrine carcinoma (NEC), which is composed of small-cell or large-cell carcinoma and mixed neuroendocrine-nonneuroendocrine neoplasms (MiNEN), which are composed of NEC and nonneuroendocrine neoplasm elements, with each component accounting for at least 30% of this cancer [2]. MiNEN or NEC of the colon usually shows aggressive progression, and patients generally have a poor prognosis [3]. However, no treatment guidelines have been established, despite conferring a worse prognosis than that of colorectal adenocarcinoma [1, 3].

We report a case of a 45-year-old man who had MiNEN of the colon with metastases to multiple organs, including the thyroid gland and the liver. The patient underwent surgical resection for each lesion, combined with systemic chemotherapy, resulting in ongoing disease-free survival for seven years. We should keep in mind that surgical extirpation of resectable tumors with systemic chemotherapy may yield long-term disease-free survival in patients with MiNEN of the colon metastasizing to multiple organs, although surgery alone has been reported to have no survival benefit for the majority of high-grade neuroendocrine neoplasms of the colon with metastatic disease [4].

## 2. Case Presentation

**Figure 1:**
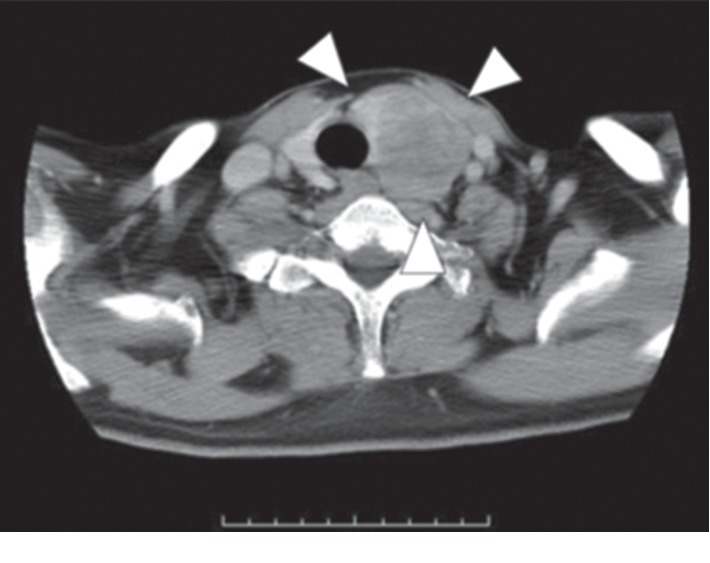
Imaging studies before the thyroidectomy. Contrast-enhanced CT showed a tumor in the left lobe of the thyroid.

**Figure 2:**
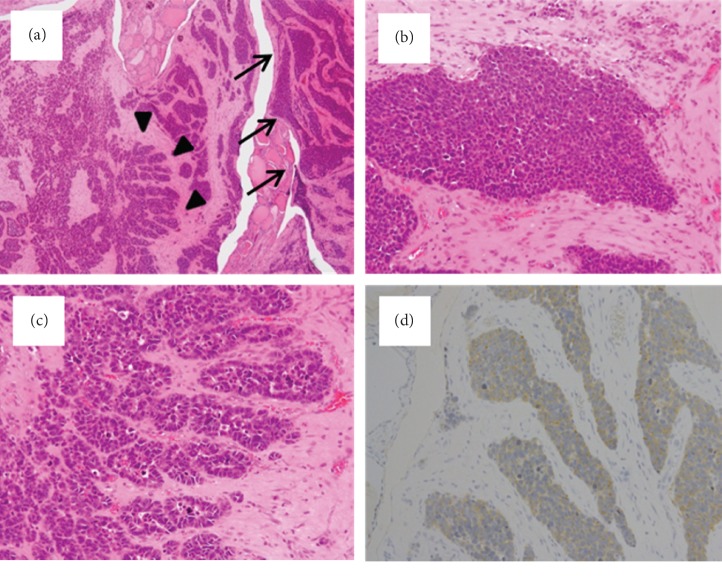
Histopathological and immunohistochemical findings of the thyroid tumor following the thyroidectomy. (A) The tumor consists of two distinct areas: a small-cell carcinoma indicated by arrows and a large-cell carcinoma shown by arrow heads (H&E staining at low power). (B) Half of the tumor shows solid growth of small cells with round-to-oval nuclei and scant cytoplasm, consistent with small-cell carcinoma (H&E staining at high power). (C) The other half shows nests and rosettes of polygonal cells with enlarged round-to-oval and prominent nuclei, consistent with large-cell carcinoma (H&E staining at high power). (D) Immunohistochemistry revealed positive staining for synaptophysin in the overall tumor at low power.

**Figure 3:**
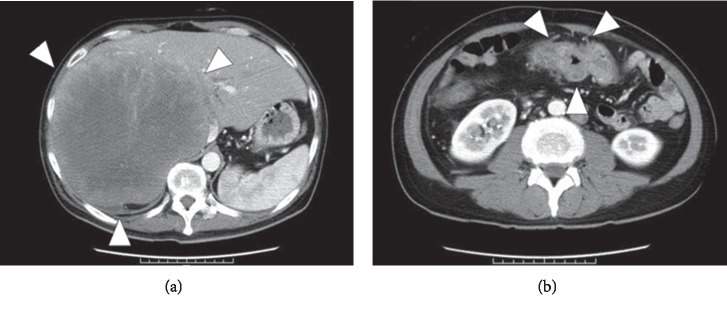
Contrast-enhanced CT scan to determine the primary site of neuroendocrine carcinoma of the thyroid. Tumors were found in the liver (A) and transverse colon (B).

**Figure 4:**
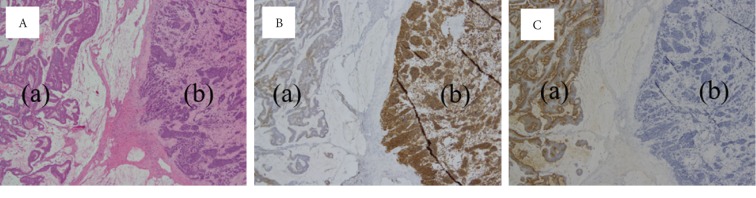
Histopathological and immunohistochemical findings of the resected transverse colon tumor. (A) The tumor was composed of well-differentiated adenocarcinoma (a) with poorly differentiated neuroendocrine carcinoma (b) and H&E staining at low power. (B) Immunohistochemistry showed that synaptophysin was partially positive in the adenocarcinoma component (a) and positive in the NEC component (b) at low power. (C) Immunohistochemistry showed that CEA was positive in the adenocarcinoma component (a) but negative in the NEC component (b) at low power.

A 45-year-old man presented to our department with rapidly progressive swelling of the anterior neck. He had been a 40-pack-year smoker. Contrast-enhanced computed tomography (CT) showed a 4.5 cm tumor that was enhanced slightly in the left lobe of the thyroid (Figure 1). At first, fine-needle aspiration cytology of the thyroid tumor suggested anaplastic carcinoma. Thereafter, we performed a left lobectomy of the thyroid to prevent fatal airway obstruction from the rapid tumor growth and to make a histological diagnosis. However, pathological examination of the thyroid tumor revealed two distinct neoplasia types (Figure 2(A)): half of the tumor showed solid growth of small cells with round-to-oval nuclei and scant cytoplasm (Figure 2(B)), and the other half was composed of nests and rosettes of polygonal cells with enlarged round-to-oval and prominent nuclei (Figure 2(C)). The specimen stained positively for synaptophysin, CD 56, and chromogranin A via immunohistochemistry (Figure 2(D)). These findings were consistent with NEC composed of small-cell and large-cell carcinoma [2], which is generally an indicator of metastasis from another organ. A whole-body CT performed to identify a primary NEC revealed a 7 cm tumor in the liver and a 3 cm tumor in the transverse colon (Figure 3); however, no tumors were detected in the lung. Ultrasound-guided needle biopsy revealed that the liver tumor was histopathologically the same type as that of the thyroid tumor, while the transverse colon tumor was found to be an adenocarcinoma based on colonoscopic biopsy. We performed colon cancer resection to prevent intestinal obstruction as palliative care. Surprisingly, two distinct areas of histological features were found in the colon specimen: well-differentiated adenocarcinoma and NEC similar to that in the thyroid and the liver (Figure 4). In addition, immunohistochemistry of the entire specimen revealed that synaptophysin and CD5 were positive in the NEC component and partially positive in the adenocarcinoma component. These findings suggested that the colon tumor was MiNEN with the adenocarcinoma and NEC components derived from the same cell of origin and the NEC component of the colon MiNEN metastasized to the thyroid and the liver [5, 6].

Chemotherapy was planned for the residual metastatic liver tumor based on a regimen for small-cell lung cancer: six cycles of chemotherapy with carboplatin and irinotecan as the first line, six cycles of chemotherapy with cisplatin and etoposide as the second line, and eight cycles of amrubicin as the third line. Disease progression was observed after the third line, which required an extensive right hepatic lobectomy and resection of the inferior vena cava. The elevated serum level of NSE and ProGRP (120 ng/ml and 59.8 pg/ml, respectively) returned to normal after hepatic lobectomy. The patient showed no evidence of disease recurrence for 7 years of follow-up after the hepatic surgery.

## 3. Discussion

In 2017, the WHO classified high-grade colorectal tumors into three categories according to the proportions of neuroendocrine and exocrine components as follows: (1) NEC (>30% neuroendocrine component and <30% nonneuroendocrine component), (2) MiNEN (>30% of both components), and (3) adenocarcinoma with neuroendocrine differentiation (>30% adenocarcinoma and <30% neuroendocrine component) [2]. Among them, NEC and MiNEN are extremely rare, accounting for only 2.1% of all cases of colorectal carcinoma [1]. Therefore, no treatment guidelines have been established for this disease, despite conferring a worse prognosis than that of colorectal adenocarcinoma [1, 3].

Cisplatin-based [4, 7, 8] or 5-FU-based regimens [3, 9] are usually regarded as standard chemotherapy for colorectal NEC and MiNEN [4, 7, 8], although chemotherapy alone is not definitive especially for patients with metastatic disease. Our chemotherapy regimen was based on the standard regimens for small-cell lung cancer including platinum agents [10]. Patta and Fakih also administered chemotherapy based on small-cell lung cancer regimens for eight patients with colon MiNEN with metastases to other organs. They reported that the median survival was only 9.5 months [11]. Recently, randomized controlled trials have demonstrated that somatostatin analogs, such as octreotide and lanreotide, prolonged progression-free survival in patients with metastatic neuroendocrine tumors [12, 13].

Some studies regard the role of primary tumor resection at an early stage as important [4, 7]; however, there have been no studies assessing the efficacy of surgery for patients with NEC or MiNEN in an advanced stage. In several studies, prolonged survival has been achieved in some patients with progressive disease by performing locoregional treatment including surgery [7, 9]. In the studies, >7 years of ongoing disease-free survival was reported in one patient with NEC of the colon with metastases to the liver who underwent hemicolectomy and repeated ablation for the liver lesion combined with chemotherapy [7]. In the present case, the primary MiNEN in the colon and the metastatic NEC in the thyroid gland and liver were all removed by surgery. Aside from surgery, the patient underwent three lines of chemotherapy based on the regimen for small-cell lung cancer [10] in accordance with other case reports of colon NEC [7, 8, 14]. As a result, long-term survival was achieved similarly to the patient mentioned by Power et al. [7]. This suggests that surgical extirpation of all resectable tumors combined with systemic chemotherapy may improve overall survival of patients with metastatic MiNEN of the colon.

Metastasis to the thyroid gland from nonthyroid sites is uncommon; however, the most common site is the lung [15–17]. In addition, the lung is the most common site for primary neuroendocrine tumors [15, 18–20]. Therefore, we first investigated the lung with CT to identify a primary lung lesion. To the best of our knowledge, this is the first case with MiNEN of the colon with metastases to the thyroid gland. When MiNEN of the colon metastasizes to another organ, the metastatic tumor usually consists of only the NEC components [14]. Therefore, digestive organ screening with CT or endoscopy is necessary to identify a primary lesion of MiNEN in the colon for patients with metastatic thyroid NEC.

Further studies are needed to establish standard guidelines for a therapeutic strategy including surgery, chemotherapy, and target therapy for patients with MiNEN of the colon.

## References

[1] Shafqat H., Ali S., Salhab M., Olszewski A. J. (2015). Survival of patients with neuroendocrine carcinoma of the colon and rectum: a population-based analysis. *Diseases of the Colon & Rectum*.

[2] Klöppel G C. A., Hruban R. H., Klimstra D. S., Komminoth P., Osamura R. Y., Lloyd RV O. R., Klöppel G., Rosai J. (2017). Neoplasms of the neuroendocrine pancreas; in: WHO classification of tumours of the endocrine organs. *WHO Classification of Tumours of Endocrine Organs*.

[3] Watanabe J., Suwa Y., Ota M. (2016). Clinicopathological and prognostic evaluations of mixed adenoneuroendocrine carcinoma of the colon and rectum: a case-matched study. *Diseases of the Colon & Rectum*.

[4] Smith J. D., Reidy D. L., Goodman K. A., Shia J., Nash G. M. (2014). A retrospective review of 126 high-grade neuroendocrine carcinomas of the colon and rectum. *Annals of Surgical Oncology*.

[5] Vortmeyer A. O., Lubensky I. A., Merino M. J. (1997). Concordance of genetic alterations in poorly differentiated colorectal neuroendocrine carcinomas and associated adenocarcinomas. *JNCI Journal of the National Cancer Institute*.

[6] de Mestier L., Cros J., Neuzillet C. (2017). Digestive system mixed neuroendocrine-non-neuroendocrine neoplasms. *Neuroendocrinology*.

[7] Power D. G., Asmis T. R., Tang L. H., Brown K., Kemeny N. E. (2011). High-grade neuroendocrine carcinoma of the colon, long-term survival in advanced disease. *Medical Oncology*.

[8] Bernick P. E., Klimstra D. S., Shia J. (2004). Neuroendocrine carcinomas of the colon and rectum. *Diseases of the Colon & Rectum*.

[9] Komatsubara T., Koinuma K., Miyakura Y. (2016). Endocrine cell carcinomas of the colon and rectum: a clinicopathological evaluation. *Clinical Journal of Gastroenterology*.

[10] Spigel D. R. (2012). Treatment update in small-cell lung cancer: from limited to extensive disease. *Current Treatment Options in Oncology*.

[11] Patta A., Fakih M. (2011). First-line cisplatin plus etoposide in high-grade metastatic neuroendocrine tumors of colon and rectum (MCRC NET): review of 8 cases. *Anticancer Research*.

[12] Rinke A., Muller H. H., Schade-Brittinger C. (2009). Placebo-controlled, double-blind, prospective, randomized study on the effect of octreotide LAR in the control of tumor growth in patients with metastatic neuroendocrine midgut tumors: a report from the PROMID Study Group. *Journal of Clinical Oncology*.

[13] Caplin M. E., Pavel M., Cwikla J. B. (2014). Lanreotide in metastatic enteropancreatic neuroendocrine tumors. *New England Journal of Medicine*.

[14] Vanacker L., Smeets D., Hoorens A. (2014). Mixed adenoneuroendocrine carcinoma of the colon: molecular pathogenesis and treatment. *Anticancer Research*.

[15] Papi G., Fadda G., Corsello S. M. (2007). Metastases to the thyroid gland: prevalence, clinicopathological aspects and prognosis: a 10‐year experience. *Clinical Endocrinology*.

[16] Wood K., Vini L., Harmer C. (2004). Metastases to the thyroid gland: the Royal Marsden experience. *European Journal of Surgical Oncology (EJSO)*.

[17] Nixon I. J., Coca-Pelaz A., Kaleva A. I. (2017). Metastasis to the thyroid gland: a critical review. *Annals of Surgical Oncology*.

[18] Beach D. F., Klump W. J., Haddad G., Reid L. M., Schwarting R., Hageboutros A. (2012). Extrapulmonary small cell: a novel case of small cell carcinoma of the thyroid gland. *Med Oncol*.

[19] Katsenos S., Archondakis S., Vaias M., Skoulikaris N. (2013). Thyroid gland metastasis from small cell lung cancer: an unusual site of metastatic spread. *Journal of Thoracic Disease*.

[20] Wey S. L., Chang K. M. (2015). Tumor-to-tumor metastasis: lung carcinoma metastasizing to thyroid neoplasms. *Case Reports in Pathology*.

